# Novel synergistic interactions between monolaurin, a mono-acyl glycerol and β lactam antibiotics against *Staphylococcus aureus*: an in vitro study

**DOI:** 10.1186/s12879-024-09261-9

**Published:** 2024-04-08

**Authors:** Shimaa Salah Hassan Abd El Ghany, Reham A. Ibrahem, Ahmed Osama EL-Gendy, Rehab Mahmoud Abd El-Baky, Ahmad Mustafa, Ahmed Farag Azmy

**Affiliations:** 1 Department of Microbiology and Immunology, Faculty of Pharmacy, Beni-Suef University, Beni-Suef, 62514 Egypt; 2 Department of Microbiology and Immunology, Faculty of Pharmacy, Deraya University, Minia, 11566 Egypt; 3 Department of Microbiology and Immunology, Faculty of Pharmacy, Minia University, Minia, 61519 Egypt; 4 Faculty of Engineering, October University for Modern Science and Arts (MSA), Giza, Egypt

**Keywords:** *S.aureus*, Monolaurin, *blaZ* gene, Gene expression, Synergism

## Abstract

**Background:**

A major worldwide health issue is the rising frequency of resistance of bacteria.Drug combinations are a winning strategy in fighting resistant bacteria and might help in protecting the existing drugs.Monolaurin is natural compound extracted from coconut oil and has a promising antimicrobial activity against *Staphylococcus.aureus*. This study aims to examine the efficacy of monolaurin both individually and in combination with β-lactam antibiotics against *Staphylococcus aureus* isolates.

**Methods:**

Agar dilution method was used for determination of minimum inhibitory concentration (MIC) of monolaurin against *S.aureus* isolates. Scanning electron microscope (SEM) was used to detect morphological changes in *S.aureus* after treatment with monolaurin. Conventional and Real-time Polymerase chain reaction (RT-PCR) were performed to detect of beta-lactamase *(blaZ)* gene and its expressional levels after monolaurin treatment. Combination therapy of monolaurin and antibiotics was assessed through fractional inhibitory concentration and time-kill method.

**Results:**

The antibacterial activity of monolaurin was assessed on 115 *S.aureus* isolates, the MIC of monolaurin were 250 to 2000 µg/ml. SEM showed cell elongation and swelling in the outer membrane of *S.aureus* in the prescence of 1xMIC of monolaurin. *bla*Z gene was found in 73.9% of *S.aureus* isolates. RT-PCR shows a significant decrease in of *blaZ* gene expression at 250 and 500 µg/ml of monolaurin. Synergistic effects were detected through FIC method and time killing curve. Combination therapy established a significant reduction on the MIC value. The collective findings from the antibiotic combinations with monolaurin indicated synergism rates ranging from 83.3% to 100%.In time-kill studies, combination of monolaurin and β-lactam antibiotics produced a synergistic effect.

**Conclusion:**

This study showed that monolaurin may be a natural antibacterial agent against *S. aureus*, and may be an outstanding modulator of β-lactam drugs. The concurrent application of monolaurin and β-lactam antibiotics, exhibiting synergistic effects against *S. aureus* in vitro, holds promise as potential candidates for the development of combination therapies that target particularly, patients with bacterial infections that are nearly incurable.

**Supplementary Information:**

The online version contains supplementary material available at 10.1186/s12879-024-09261-9.

## Introduction

Gram-positive *S.aureus* develops in clusters that resemble grapes and has a spherical form (cocci). This facultative anaerobe is frequently found on the skin, in the nose, and in the respiratory system. *S.aureus* can cause food poisoning and toxic shock syndrome in addition to skin infections like abscesses and pyogenic infections (such as endocarditis and septic arthritis), respiratory infections like sinusitis and hospital-acquired pneumonia [1]. Over the preceding decades, Antibiotic resistance has been developed as a result of widespread overprescription, self-medication, and overuse of therapeutically available antibiotics, which has precipitated prolonged exposure of pathogenic microorganisms to these antimicrobial agents [2]. The process underlying antibiotic resistance, consequent to extended exposure,involves the accumulation of several genes, each conferring resistance to a specific antibiotic. Within individual bacterial cells, this mechanism has notably facilitated the proliferation of multidrug-resistant (MDR) bacterialstrains.MDR bacteria employ horizontal gene transfer mechanisms to disseminate antibiotic resistance genes among their population [3]. Several diseases were attributed to multidrug-resistant (MDR) bacterial strains proved to be incurable and fatal owing to their elevated resistance levels against the majority of clinically accessible antibiotics. Presently, it was documented that over 70% of pathogenic bacteria have acquired such resistance [4]. Major human bacterial pathogen *S. aureus* can develop resistance to the majority of antibiotics [5]. For instance, the clinical usage of methicillin led to the emergence of methicillin-resistant *S. aureus* (MRSA) [6]. MRSA is a widespread bacterium that can cause a broad variety of infections, from minor skin irritations to serious, even life-threatening conditions including sepsis and endocarditis [7]. It puts a heavy pressure on the world's healthcare system [8]. It has been determined that *S.aureus* can resist β -lactams in two different ways. The most important step in the production of the β-lactamase enzyme, which breaks down the β -lactam ring of antibiotics, is encoded by the *bla*Z gene. In addition to its usual location on plasmids, the *blaZ* gene is also present in the chromosomal DNA of the bacteria. The two nearby genes blaI and blaR1, which serve as *bla*Z's anti-repressor and transcription repressor, respectively, control the expression of *blaZ* [9]. The development of new β-lactam type antibiotics or β-lactamase inhibitors is a hotly researched topic since lactamase-mediated antibiotic resistance is a significant public health concern [10]. In addition to using β-lactamase inhibitors, which are the most promising method, alternative tactics, are being considered to inhibit multidrug resistant (MDR) microorganisms. Antimicrobial peptides, nanoparticles, bacteriophages, various peptide nano formulations, and combinations with commercial antibiotics are some of these [11]. Throughout history, traditional medicine has frequently utilized medicinal plants or their derivatives to combat various infectious diseases. Numerous reports have highlighted the antimicrobial properties exhibited by various plants or their extracts [12]. When plant remedies are employed in conjunction with antimicrobial drugs, specific herb-drug interactions potentially yielding synergistic augmentation of antimicrobial efficacy and mitigating adverse synthetic drug effects. These synergistic effects have undoubtedly reduced the probability of diminished drug efficacy when administered alone against microbial infections over prolonged periods [13].

Moreover, the strategy of combining herbs with drugs may facilitate the discovery of novel antibiotics and the reintroduction of those antibiotics to which bacteria have developed resistance, thereby offering a promising opportunity for combating antimicrobial resistance [14]. Herbal products, such as medium-chain fatty acids and essential oils, whether employed as dietary supplements or as additives for food preservation, are recognized for their antimicrobial attributes. Monolaurin is a monoester created from lauric acid and glycerol, commonly known as glycerol monolaurate. Although lauric acid constitutes a significant proportion of virgin coconut oil, the levels of monolaurin in virgin coconut are typically low. Nevertheless, when orally ingested or utilized as a dietary supplement, certain coconut oil fractions undergo hydrolysis catalyzed by pancreatic lipase, resulting in the formation of lauric acid monoglyceride [15]. The Food and Drug Administration (FDA) usually recognizes glycerol monolaurate as safe for human use, and the cosmetic and food sectors frequently employ this substance. This substance has strong antibacterial effects on *Bacillus anthracis* and Gram-positive cocci [16]. It has been demonstrated that monolaurin works against *S.aureus* strains that are both sensitive and resistant [17]. In contrast to the majority of antibiotics, which typically target specific bacterial sites for their antibacterial effects, GML (glycerol monolaurate) seems to act on numerous bacterial surface signal transduction systems indiscriminately by interacting with plasma membranes. Furthermore, it may prove valuable as an environmental surface microbicide for controlling bacterial infections and contamination [18].

## Materials and methods

### Bacterial isolates

The study included 115 *S.aureus* strains that were obtained from different infection sites in patients admitted to different hospitals in Minia governorate, Egypt, during the period from September 2021 to April 2022 (Additional file 1). The study was approved by the Ethical Review Board of Faculty of Pharmacy, Deraya University, Minia, Egypt. Approval no. (9/2023).By using conventional laboratory techniques, isolates were identified morphologically and biochemically. *S.aureus* isolates were distinguished using the coagulase and DNase assays. The staphylococcal isolates were kept alive after identification in Trypticase soy broth (TSB), to which 15% glycerol was added, and were kept at -20 °C.

### Antibiotic susceptibility testing

Following the recommendations of the Clinical and Laboratory Standards Institute (CLSI, 2020), the antimicrobial susceptibility profile of *S.aureus* isolates was evaluated using the Kirby-Bauer disc diffusion method [19]. The following antibiotics were tested: ampicillin/sulbactam (20 µg), amoxicillin/clavulunic acid (30 µg), piperacillin/tazobactam (10 µg), gentamicin (10 µg), amikacin (30 µg), ciprofloxacin (5 µg), levofloxacin (5 µg), tetracycline (30 µg) chloramphenicol (30 µg), imipenem (10 µg), rifampin (5 µg), and linezolid (30 µg) (Oxoid, UK). Cefoxitin disc diffusion method was used for Methicillin resistant *S.aureus* (MRSA). Vancomycin susceptibility of isolates was assessed using the agar dilution method.

### Detection of blaZ gene among S.aureus isolates by PCR

On sheep blood agar plates, all isolated *S.aureus* strains were cultured for a whole night at 37 °C. At 37 °C for 24 h, one colony was kept suspended in 1 ml of LB broth (Sigma Chemical Company, St. Louis, MO). According to [20] the 115 *S.aureus* isolates had their genomic DNA extracted using a DNA extraction kit (QIAamp DNA Mini Kit) instructions. The oligonucleotide primer sequences for *blaZ* gene were 5’TACAACTGTAATATCGGAGGG’3 for forward primer and 5’CATTACACTCTT GGCGGTTT’3 for reverse primer. The polymerase chain reaction (PCR) was conducted under the following conditions: initial denaturation took place at 94 °C for 5 min, then 35 cycles of amplification were performed using the following parameters: 94 °C for 30 s, annealing at 49 °C for 40 s, extension at 72 °C for 50 s, and a final extension step at 72 °C for 10 min. Electrophoresis was used to separate the PCR products on a 1.5% agarose gel, and it was done for 30 min at a continuous current of 1–5 V/cm. Ethidium bromide staining and UV transillumination light were used to identify DNA bands. By comparing the fragments' migration to a 100 bp ladder as a reference, the size of the fragments was identified [21].

### Monolaurin preparation

For preparation of a stock solution, 4 mg of monolaurin was firstly solubilized in pure dimethyl sulfoxide (DMSO) (100 µL). Next, this stock solution was combined with 1900 µL Tryptic Soy Broth (TSB) media, to yield a final volume of 2000 µL, thereby achieving a concentration of 2 mg/ml with 5% DMSO content. Subsequent concentrations of monolaurin were derived from this initial stock solution. A concentration of 5% DMSO was used as a negative control [22].

### Determination of Minimum Inhibitory Concentrations (MICs) of monolaurin and certain β lactam antibiotics

MIC was the lowest antibacterial agent concentrations that completely stop bacterial growth for 24 h. The agar dilution method was used to assess the MICs of ampicillin, amoxicillin, piperacillin, and monolaurin for 115 *S.aureus* isolates. The Mueller–Hinton Broth (MHB) was prepared to have 10^7^ colony forming unit per milliliter (CFU/ml) of cells for overnight cultures of the tested isolates. Using routine serial two-fold dilutions, the tested antibiotics and monolaurin were added to Muller-Hinton Agar (MHA)**.** Microbial inoculum is then administered to the surface of the agar plate using a multi-inoculator [23, 24].

### Scanning electron microscope

The approach described by [25] with a few minor adjustments was used to conduct the scanning electron microscopy operation. The bacteria were taken after their overnight incubation, resuspended in fresh MHB, and treated with 1 × MIC monolaurin at 37 °C for 2 h. After incubation, cells were removed using a centrifuge (4,000 × g, 10 min) and twice-washed in 0.1 M PBS (pH 7.2). After that, bacteria were fixed for an overnight period at 48 °C using 2.5% (v/v) glutaraldehyde in 0.1 M PBS. The samples were initially dehydrated in a gradient of ethanol (30%, 50%, 80%, 90%, 95%, 100% (v/v)).Vacuum freeze-drying equipment was used to dry the samples for 8 h. Every bacterial culture was examined by SEM with accelerating voltage 0.3 to 30 kV and magnification power up from 5000 × to 300,000x (Hitachi, Japan). As a negative control, bacterial cell suspension in MHB without any medication was used.

### Gene expression of *blaZ* gene using Real-Time Polymerase Chain Reaction (RT-PCR)

To evaluate the relative expression of the *blaZ* gene, reverse transcription polymerase chain reaction (RT-PCR) was employed under varying concentrations of monolaurin (0.25XMIC and 0.5XMIC). Additional file 2 contains the primer sequences that were employed in this study. Fresh tryptic soy broth (TSB) was inoculated with overnight cultures of *S. aureus*, followed by incubation at 37 °C. Subsequently, S. aureus cultures and TSB containing sub-minimal inhibitory concentrations (sub-MIC) of monolaurin were aliquoted into test tubes. Incubation overnight at 37 °C was conducted for both experimental and control tubes. Prior to and post treatment with monolaurin, gene expression analysis for *blaZ*, normalized to the 16S rRNA housekeeping gene, was performed on the selected isolates. Total RNA extraction followed the guidelines outlined in the RNeasy Mini Kit. Cycling conditions were as previously outlined in Sect. 2.3. Gene expression levels were standardized to 16S rRNA, and amplification curves and Ct values were determined using the Stratagene MX3005P program. Measuring the variance in gene expression on the RNA of various samples by using the "Ct" approach described by Yuan et al. [26], the CT of each sample was compared to that of the control group, To rule out false positive results, dissociation curves from several samples were compared.

### Testing the effect of monolaurin and certain β-lactam antibiotics combinations using Fractional inhibitory concentration (FIC assay)

The agar dilution method was employed to test the synergy. By using the FIC assay, the effects of monolaurin combinations with the tested antibiotics at sub-MIC concentrations were evaluated against MDR *S.aureus* isolates. Ampicillin (0.25–32 mg/L in 8 two-fold dilutions), Amoxicillin(0.25–128 mg/L in 10 two-fold dilutions), Piperacillin (0.25–256 mg/L in 11two-fold dilutions) and the appropriate concentration of monolaurin (250 µg/ml) or(500 µg/ml) were added to medium separetly. The bacterial strains were diluted from an overnight broth, to give an inoculum of 10^4^ cfu per spot when applied with a Multipoint Inoculator. Inhibition was read after incubation for 24 h at 37 °C,

To evaluate the effect of combination, The FIC index values were then calculated using the following formula: ƩFICI = FIC (A) + FIC (B)

$$\mathrm{where\, FIC\, }({\text{A}}) = \frac{\mathrm{MIC }\,({\text{A}})\,\mathrm{ in\, combination}}{\mathrm{\,MIC\, }({\text{A}})\mathrm{\, alone}}\mathrm{\, and\, FIC\, }({\text{B}}) =\frac{\mathrm{\,MIC\, }({\text{B}}\,)\mathrm{\, in\, combination}}{\mathrm{MIC\, }({\text{B}})\mathrm{\, alone}}.$$

The ∑ FICI values were interpreted as follows: total synergistic ∑FIC ≤ 0.5, partial synergism (0.5 < ∑FIC < 1), additive, ∑FIC = 1, indifference 1 < ∑FIC < 4 and antagonistic (∑FIC ≥ 4) [27, 28].

### Time-kill assay

Both monolaurin alone and in combination with piperacillin, amoxicillin, and ampicillin were studied. Their concentrations were in the 0.25 to 0.5 MIC range. Control tests lacking antibacterial substances. The vials were incubated at 37 °C with cation-adjusted Mueller–Hinton broth, antimicrobials, and the tested organisms at an initial density of 10^6^ CFU/ml (10 ml volume). A viable-colony count was performed by serially diluting aliquots at 0, 4, 6, 8, 12 and 24 h before plating them on Mueller–Hinton agar plates. After 24 h of incubation, the synergy effect was defined as a ≥ 2 log_10_ CFU/ml decrease in colony counts as compared to the single agent with the highest activity. The antagonism was defined by an increase of ≥ 2 log_10_ CFU/ml in the combination compared to the most active single agent. Colony counting with an antibiotic combination against separate antimicrobials results in an increase or decrease of 2 log_10_, which was defined as the no difference (ND) impact [29].

### Statistical analysis

The SPSS 25.0 programme was used to analyse the data. The Kolmogorov–Smirnov test was used to determine whether the distribution was normal or not. Microbiological analysis outcomes showed a non-parametric distribution. Non-parametric data was measured using the Mann–Whitney U-test. The results were evaluated using a statistical test for paired non-parametric (Wilcoxon and Friedman) samples.

## Results

### Resistance pattern of *S.aureus* isolates

**Fig. 1:**
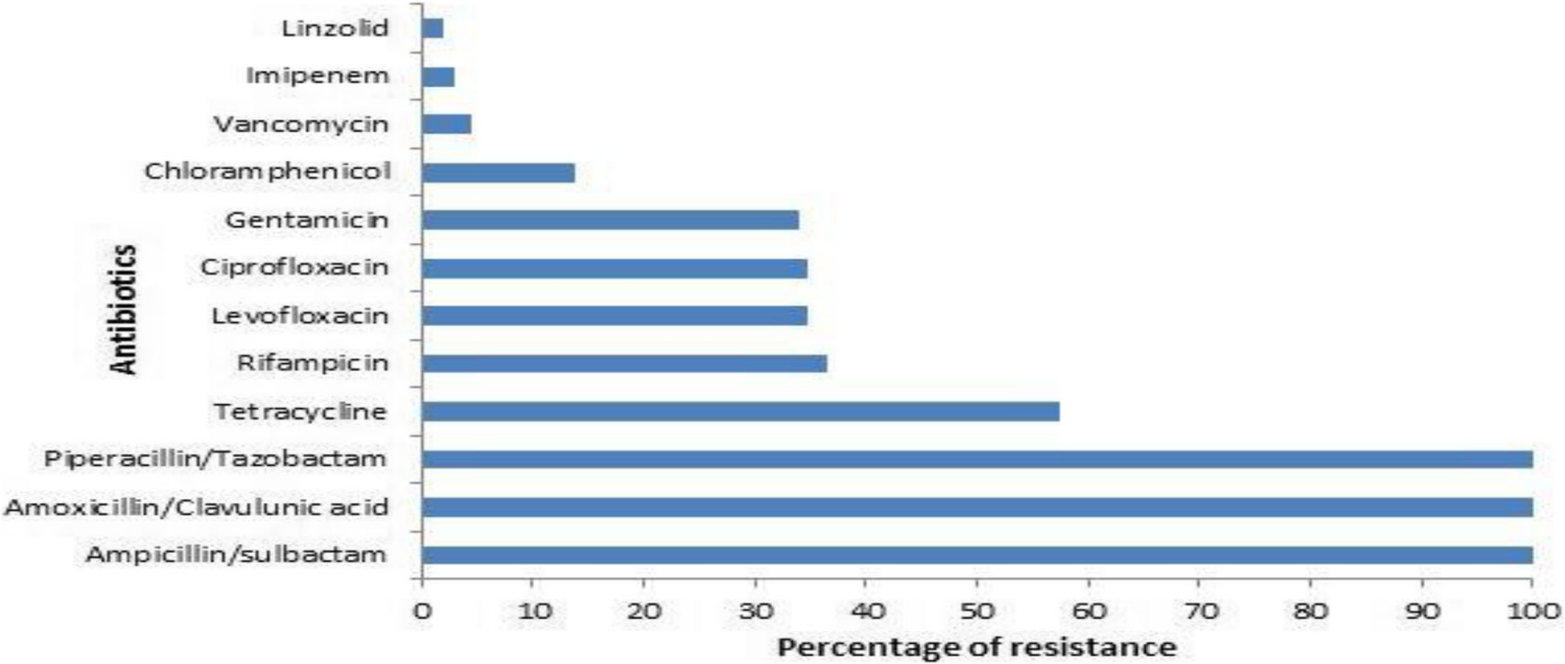
The resistance pattern of the isolated *S.aureus* strains

Figure 1 determines the resistance pattern of *S.aureus* strains. Regarding *S.aureus* isolates, they revealed complete resistance to ampicillin/sulbactam, amoxicillin/clavulunic acid and piperacillin/tazobactam (100%), high resistance against tetracycline (57.4%), moderate resistance against rifampicin (36.52%), ciprofloxacin (34.8%), levofloxacin (34.8%) and gentamicin (33.9%) and low resistance against chloramphenicol (13.9%), vancomycin (4.35%), imipenem (3%), and linezolid (2%). Cefoxitin was used to determine MRSA. Out of *S.aureus* isolates, 103 (89.6%) were MRSA and 12 (10.4%) were MSSA.

### Molecular detection of *blaZ* gene by conventional PCR

**Fig. 2:**
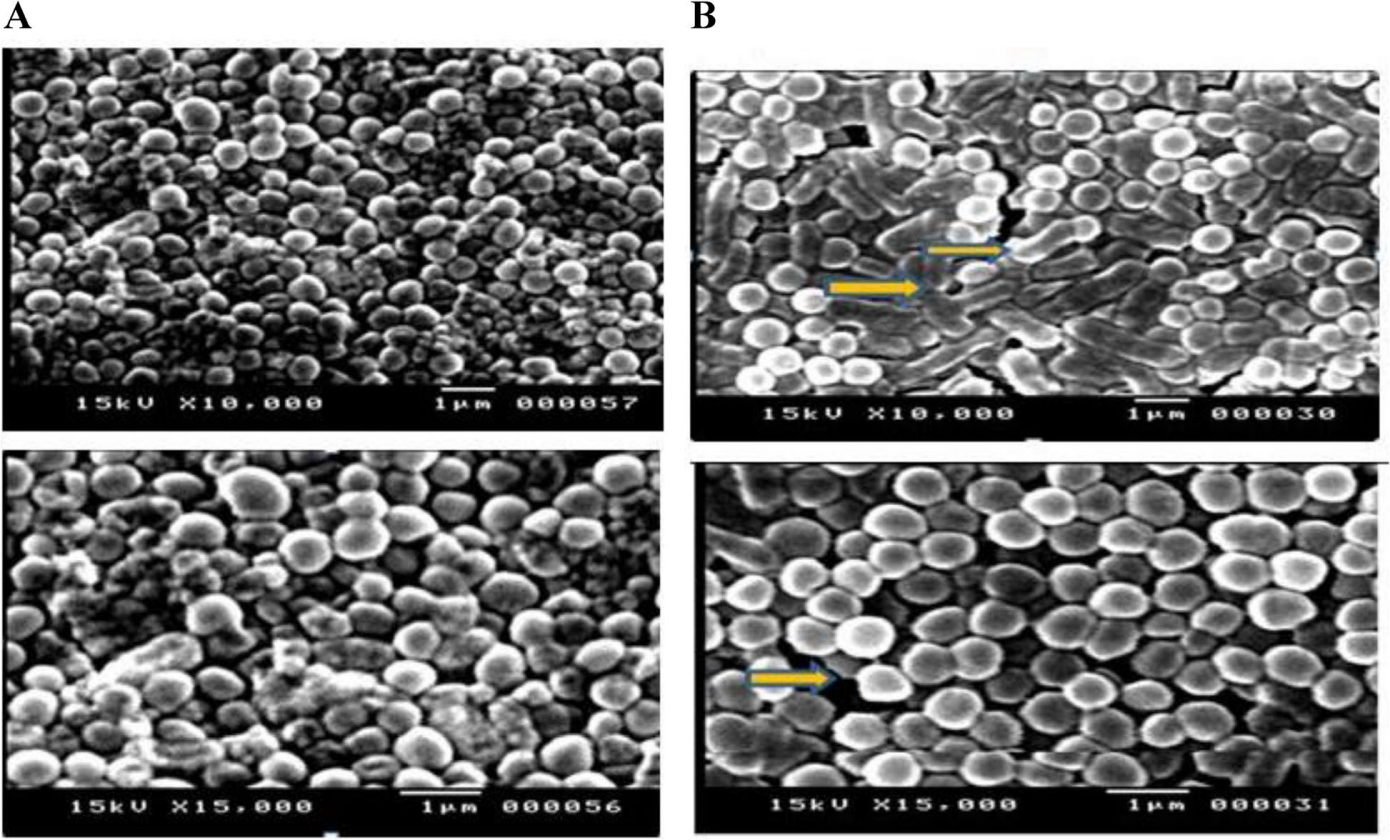
Effect of monolaurin on *S.aureus* by SEM under 10000X and 15,000 X magnifications; **A** Control culture of *S.aureus* and (**B**) *S.aureus* treated with 1000 µg/ml of monolaurin

Out of 115 *S.aureus* isolates 85 (73.9%) *S.aureus* isolates harboured *blaZ* gene. *blaZ* showed PCR product at 833 bp as illustrated in Fig. 2 (Additional file 3).

### Determination of minimum inhibitory concentration

The MIC of monolaurin was determined against 12 MSSA and 103 MRSA as shown in Table 1, the MIC range of monolaurin ranged from 500 to1000 µg/mL for MSSA and from 250 to 2000 µg/mL for MRSA. A concentration of 5% DMSO, did not exhibit any noticeable effect on bacterial growth.

**Table 1:** MICs of Monolaurin against clinical isolates of MSSA and MRSA strains

Monolaurin	MIC against MSSA	No.of isolates	%*	MIC against MRSA	No.of isolates	%*
	500	2	1.7	250	3	2.6
				500	9	7.8
	1000	10	8.7	1000	83	72.2
				2000	8	7
**Total No.of isolates**	115	12	10.4		103	89.6

%* was correlated to total number of *S.aureus* isolates (no=115)

### Scanning electron microscope

SEM analysis supported the impact of monolaurin on *S.aureus*'s cell structure. Treated cells with the tested monolaurin at a concentration of 1xMIC (1000 µg/ml) showed a change in morphology in the form of cell elongation and swelling when compared to the control while untreated bacteria were intact (regular cocci-shaped).The examined bacteria had severe structural changes in the outer membrane of *S.aureus*, leading to cell death. Monolaurin changed the cellular structure and outer membrane as illustrated in Fig. 2.

### Effect of monolaurin on expression of blaZ gene among S.aureus strains

**Fig. 3:**
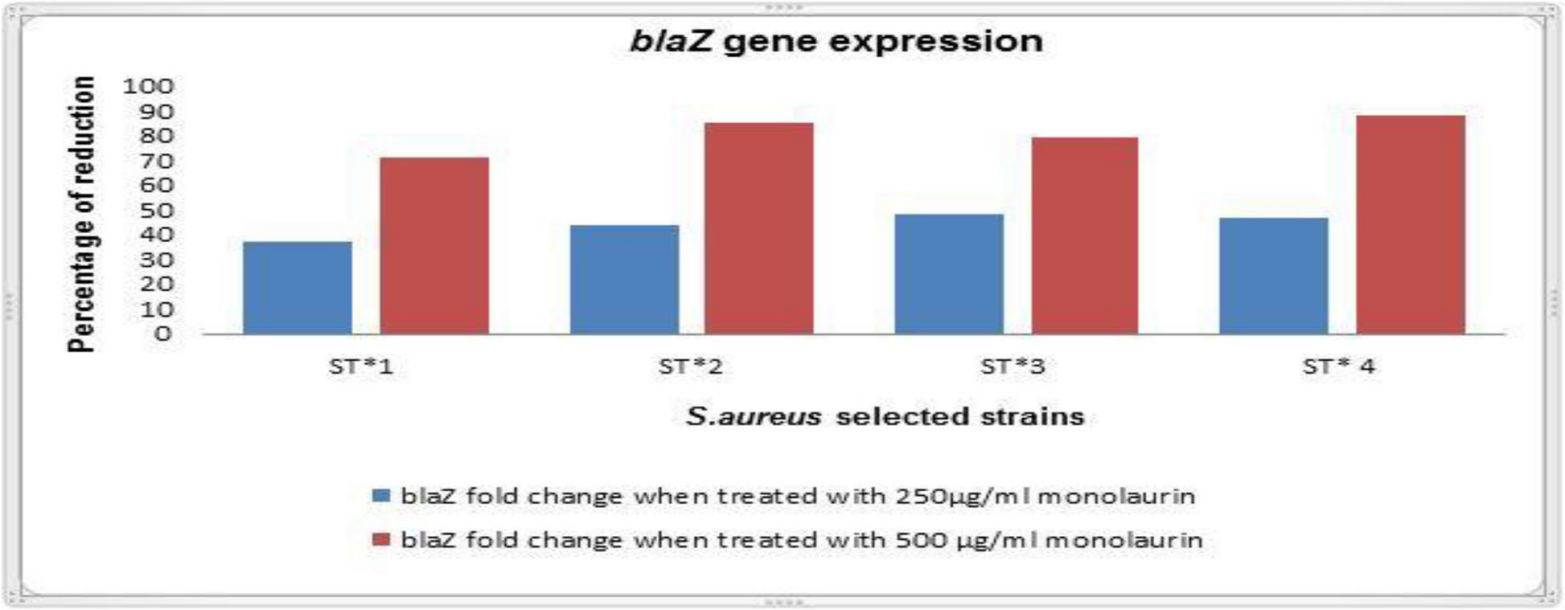
Effect of monolaurin on expression of *blaZ* gene among *S.aureus* strains. ST*,* Staphylococcus aureus*

The results showed that the expression level of *blaZ* gene was down regulated as shown in Fig. 3. The *blaZ* gene exhibited no fold change in control samples. Four isolates were chosen for testing the activity of monolaurin in reduction of gene expression of *blaZ* gene (Additional files 4 and 5). Upon using 250 µg/ml monolaurin on the tested strains, a fold decrease in the expression of *blaZ* gene ranging from 37.15–47.15 while using 500 µg/ml monolaurin there was a 71.48–88.09 fold reduction (Additional file 6).

### Synergistic effect of tested antibiotics and monolaurin

MICs of the tested Antibiotics as Monotherapy and in Combination with monolaurin.

The effectiveness of monolaurin when combined with β-lactam antibiotics (ampicillin, amoxicillin, and piperacillin) was tested using the agar dilution technique against MRSA and MSSA isolates. For MRSA isolates, the MIC of Ampicillin was 8-32 µg/ml when tested alone; the MIC decreased to 1–4 µg/ml (4–32 fold reduction, *p* < 0.001),The MIC of Amoxicillin was 32-128 µg/ml when tested alone; the MIC decreased to 0.5–8 µg/ml (4–128 fold reduction,* p* < 0.001).The MIC of piperacillin was 16-256 µg/ml when tested alone; the MIC decreased to 4–32 µg/ml (2–32 fold reduction,* p* < 0.001) when combined with 250 µg/ml monolaurin (Table 2). The MIC of Ampicillin was 8-32 µg/ml when tested alone; the MIC decreased to 0.5–2 µg/ml (8–64 fold reduction, *p* < 0.001),The MIC of Amoxicillin was 32-128 µg/ml when tested alone; the MIC decreased to 0.5–4 µg/ml (8–256 fold reduction,* p* < 0.001)The MIC of piperacillin was 16-256 µg/ml when tested alone; the MIC decreased to 0.5–16 µg/ml (8–256 fold reduction,* p* < 0.001) when combined with 500 µg/ml monolaurin (Table 3).

**Table 2:** Reduction of MICs of β-lactam antibiotics combined with Monolaurin (250 µg/ml) against MRSA (*n* = 103)

MIC (µg/mL)		FICA/FICB	FIC index	Interpretation	No. of isolates	*P* value
**Alone**	**In combination**					
**Ampicillin /Monolaurin**	**Ampicillin /Monolaurin**					
8/1000	1/250	0.125/0.25	**0.375**	**Synergistic**	**15**	** < 0.001**
8/250	1/250	0.125/1	**1.125**	**Indifference**	**3**	
8/1000	2/250	0.25/0.25	**0.5**	**Synergistic**	**20**	
16/500	1/250	0.0625/0.5	**0.5625**	**Partial synergy**	**9**	
16/1000	4/250	0.25/0.25	**0.5**	**Synergistic**	**7**	
32/1000	1/250	0.03125/0.25	**0.281**	**Synergistic**	**16**	
32/1000	4/250	0.125/0.25	**0.375**	**Synergistic**	**22**	
32/2000	1/250	0.03125/0.125	**0.1562**	**Synergistic**	**3**	
32/2000	4/250	0.125/0.125	**0.25**	**Synergistic**	**8**	
**Amoxicillin (A) /Monolaurin (B)**	**Amoxicillin (A) /Monolaurin (B)**	**FIC (A) / FIC (B)**	**FIC index**	**Interpretation**	**No. of isolates**	***P* value**
32/250	0.5/250	0.015625/1	**1.015**	**Indifference**	**3**	** < 0.001**
32/1000	0.5/250	0.015625/0.25	**0.265**	**Synergistic**	**3**	
32/1000	4/250	0.125/0.25	**0.375**	**Synergistic**	**2**	
32/500	4/250	0.125/0.5	**0.625**	**Partial synergy**	**9**	
32/1000	8/250	0.25/0.25	**0.5**	**Synergistic**	**12**	
64/1000	0.5/250	0.0078/0.25	**0.2578**	**Synergistic**	**32**	
64/1000	2/250	0.03125/0.25	**0.281**	**Synergistic**	**5**	
64/1000	4/250	0.0625/0.25	**0.3125**	**Synergistic**	**16**	
64/1000	8/250	0.125/0.25	**0.375**	**Synergistic**	**11**	
128/1000	1/250	0.0078/0.25	**0.2578**	**Synergistic**	**2**	
128/2000	1/250	0.0078/0.125	**0.1328**	**Synergistic**	**4**	
128/2000	8/250	0.0625/0.125	**0.1875**	**Synergistic**	**4**	
**Piperacillin /Monolaurin**	**Piperacillin /Monolaurin**	**FIC (A) / FIC (B)**	**FIC index**	**Interpretation**	**No. of isolates**	***P* value**
16/250	4	0.25/1	**1.25**	**Indifference**	2	** < 0.001**
16/250	8	0.5/1	**1.5**	**Indifference**	1	
16/1000	8	0.5/0.25	**0.75**	**Partial synergy**	17	
64/1000	16	0.25/0.25	**0.5**	**Synergistic**	12	
64/500	8/250	0.0625/0.5	**0.5625**	**Partial synergy**	9	
128/1000	16	0.125/0.25	**0.375**	**Synergistic**	20	
128/1000	32	0.25/0.25	**0.5**	**Synergistic**	16	
256/1000	32/250	0.125/0.25	**0.375**	**Synergistic**	14	
256/1000	8/250	0.03125/0.25	**0.28125**	**Synergistic**	4	
256/2000	8/250	0.03125/0.125	**0.15625**	**Synergistic**	8	

^*^^total synergistic (FICI≤0.5), partial synergistic (0.5 < FICI < 1), additive FICI=1 or indifferent (1 < FICI < 4 and antagonistic (FICI≥4)^

^**significant difference at P value < 0.05^

**Table 3:** Reduction of MICs of β-lactam antibiotics combined with Monolaurin (500 µg/ml) against MRSA (*n* = 103)

MIC (µg/mL)		FIC (A) / FIC (B)	FIC index	Interpretation	No. of isolates	*P* value
**Alone**	**In combination**					
**Ampicillin (A) /Monolaurin(B)**	**Ampicillin (A) /Monolaurin (B)**					
8/1000	0.5/500	0.0625/0.5	**0.56**	**Partial synergy**	24	** < 0.001**
8/250	0.5/500	0.062/2	**2.062**	**Indifference**	3	
8/500	2/500	0.25/1	**1.25**	**Indifference**	9	
8/1000	2/500	0.25/0.5	**0.75**	**Partial synergy**	2	
16/1000	0.5/500	0.03125/0.5	**0.53**	**Partial synergy**	28	
16/1000	2/500	0.125/0.5	**0.625**	**Partial synergy**	4	
32/1000	0.5/500	0.015625/0.5	**0.515**	**Partial synergy**	22	
32/1000	2/500	0.0625/0.5	**0.5625**	**Partial synergy**	3	
32/2000	2/500	0.0625/0.125	**0.1875**	**Synergistic**	8	
**Amoxicillin /Monolaurin**	**Amoxicillin /Monolaurin**	**FICA/FICB**	**FIC index**	**Interpretation**	**No.of isolates**	***P* value**
32/250	0.5/500	0.015625/2	**2.015**	**Indifference**	**3**	** < 0.001**
32/1000	0.5/500	0.015625/0.5	**0.515**	**Partial synergy**	**20**	
32/1000	4/500	0.125/0.5	**0.625**	**Partial synergy**	**6**	
64/500	0.5/500	0.0078/1	**1.0078**	**Indifference**	**9**	
64/1000	0.5/500	0.0078/0.5	**0.5078**	**Partial synergy**	**44**	
64/1000	1/500	0.015625/0.5	**0.515**	**Partial synergy**	**5**	
64/1000	4/500	0.0625/0.5	**0.5625**	**Partial synergy**	**6**	
128/1000	0.5/500	0.0039/0.5	**0.5039**	**Partial synergy**	**2**	
128/2000	0.5/500	0.0039/0.25	**0.2539**	**Synergistic**	**8**	
**Piperacillin /Monolaurin**	**Piperacillin /Monolaurin**	**FICA/FICB**	**FIC index**	**Interpretation**	**No. of isolates**	***P* value**
16/250	1/500	0.0625/2	**2.0625**	**Indifference**	2	** < 0.001**
16/250	0.5/500	0.03125/2	**2.03125**	**Indifference**	1	
16/1000	0.5/500	0.03125/0.5	**0.53125**	**Partial synergy**	25	
64/500	0.5/500	0.0078/1	**1.0078**	**Indifference**	5	
64/500	1/500	0.0156/1	**1.0156**	**Indifference**	4	
128/1000	1/500	0.0078/0.5	**0.5078**	**Partial synergy**	16	
128/1000	8/500	0.0625/0.5	**0.5625**	**Partial synergy**	12	
128/1000	0.5/500	0.0039/0.5	**0.5039**	**Partial synergy**	12	
128/1000	1/500	0.0078/0.5	**0.5078**	**Partial synergy**	6	
128/1000	16/500	0.125/0.5	**0.625**	**Partial synergy**	12	
256/2000	8/500	0.03125/0.25	**0.28**	**Synergistic**	8	

^*^^total synergistic (FICI≤0.5), partial synergistic (0.5 < FICI < 1), additive FICI=1 or indifferent (1 < FICI < 4 and antagonistic (FICI≥4)^

^**significant difference at*P*value < 0.05^

For MSSA isolates, the MIC of Ampicillin was8-16 µg/ml when tested alone; the MIC decreased to 1-4 µg/ml (4–16 fold reduction, *p* < 0.001),The MIC of Amoxicillin was 32-64 µg/ml when tested alone; the MIC decreased to 0.5–2 µg/ml (16–128 fold reduction, *p* < 0.001).The MIC of piperacillin was 16-256 µg/ml when tested alone; the MIC decreased to2-32 µg/ml (2–eightfold reduction, *p* < 0.001) when combined with 250 µg/ml monolaurin (Table 4). The MIC of Ampicillin was 8-16 µg/ml when tested alone; the MIC decreased to0.5–1 µg/ml (16–32 fold reduction, *p* < 0.001), the MIC of Amoxicillin was 32–64 µg/ml when tested alone; the MIC decreased to0.5 µg/ml (64–128 fold reduction, *p* < 0.001)The MIC of piperacillin was 16-256 µg/ml when tested alone; the MIC decreased to 0.5–8 µg/ml (32–256 fold reduction, *p* < 0.001) when combined with 500 µg/ml monolaurin (Table 5).

**Table 4:** Reduction of MICs of β-lactam antibiotics combined with Monolaurin (2500 µg/ml) against MSSA (*n* = 12)

MIC (µg/mL)		FIC (A) / FIC (B)	FIC index	Interpretation	No. of isolates	*P* value
**Alone**	**In combination**					
**Ampicillin (A) /Monolaurin(B)**	**Ampicillin (A) /Monolaurin (B)**					
8/1000	1/250	0.125/0.25	**0.375**	**Synergistic**	3	** < 0.001**
8/500	2/250	0.25/0.5	**0.75**	**Partial synergy**	2	
16/1000	1/250	0.0625/0.25	**0.3125**	**Synergistic**	4	
16/1000	4/250	0.25/0.25	**0.5**	**Synergistic**	3	
**Amoxicillin /Monolaurin**	**Amoxicillin /Monolaurin**	**FICA/FICB**	**FIC index**	**Interpretation**	**No.of isolates**	***P* value**
32/1000	0.5/250	0.015625/0.25	**0.2656**	**Synergistic**	3	** < 0.001**
32/1000	2/250	0.0625/0.25	**0.3125**	**Synergistic**	4	
64/500	0.5/250	0.0078/0.5	**0.5078**	**Partial synergy**	2	
64/1000	2/250	0.03125/0.25	**0.28125**	**Synergistic**	3	
**Piperacillin /Monolaurin**	**Piperacillin /Monolaurin**	**FICA/FICB**	**FIC index**	**Interpretation**	**No. of isolates**	***P* value**
16/1000	2/250	0.125/0.25	**0.375**	**Synergistic**	1	** < 0.001**
16/1000	4/250	0.25/0.25	**0.5**	**Synergistic**	6	
16/500	8/250	0.5/0.5	**1**	**Additive**	2	
128/1000	16/250	0.125/0.25	**0.375**	**Synergistic**	1	
256/1000	32/250	0.125/0.25	**0.375**	**Synergistic**	2	

^*^^total synergistic (FICI≤0.5), partial synergistic (0.5 < FICI < 1), additive FICI=1 or indifferent (1 < FICI < 4) and antagonistic (FICI≥4) **significant difference at P value < 0.05^

**Table 5:** Reduction of MICs of β-lactam antibiotics combined with Monolaurin (500 µg/ml) against MSSA (*n* = 12)

MIC (µg/mL)		FIC (A) / FIC (B)	FIC index	Interpretation	No. of isolates	*P* value
**Alone**	**In combination**					
**Ampicillin (A) /Monolaurin(B)**	**Ampicillin (A) /Monolaurin (B)**					
8/500	0.5/500	0.0625/1	**1.0625**	**Indifference**	2	** < 0.001**
8/1000	0.5/500	0.0625/0.5	**0.5625**	**Partial synergy**	3	
16/1000	0.5/500	0.031/0.5	**0.531**	**Partial synergy**	4	
16/1000	1/500	0.0625/0.5	**0.5625**	**Partial synergy**	3	
**Amoxicillin /Monolaurin**	**Amoxicillin /Monolaurin**	**FICA/FICB**	**FIC index**	**Interpretation**	**No. of isolates**	***P* value**
32/500	0.5/500	0.015625/1	**1.0156**	**Indifference**	2	** < 0.001**
32/1000	0.5/500	0.015625/0.5	**0.515625**	**Partial synergy**	5	
64/1000	0.5/500	0.0078/0.5	**0.5078**	**Partial synergy**	5	
**Piperacillin /Monolaurin**	**Piperacillin /Monolaurin**	**FICA/FICB**	**FIC index**	**Interpretation**	**No. of isolates**	***P* value**
16/500	0.5/500	0.031/1	**1.031**	**Indifference**	2	** < 0.001**
16/1000	0.5/0.5	0.031/0.5	**0.531**	**Partial synergy**	7	
128/1000	1/500	0.0078/0.5	**0.5078**	**Partial synergy**	1	
256/1000	1/500	0.0039/0.5	**0.5039**	**Partial synergy**	1	
256/1000	8/500	0.031/0.5	**0.531**	**Partial synergy**	1	

^*^^total synergistic (FICI≤0.5), partial synergistic (0.5 < FICI < 1), additive FICI=1 or indifferent (1 < FICI < 4) and antagonistic (FICI≥4) **significant difference at*P*valuse < 0.05^

Synergy testing results for three combinations against MRSA and MSSA isolates.

For MRSA isolates, the combination of 250 µg/ml monolaurin with ampicillin, amoxicillin and piperacillin exhibited synergism in 88.4%, 88.4% and 71.8%, partial synergy in 8.7%, 8.7% and 25.3%and indifference in 2.9% in the three combinations, respectively. Using 500 µg/ml monolaurin with ampicillin, amoxicillin and piperacillin showed synergism in 7.8%, partial synergy 80.6% and indifference in 11.6% of the total isolates in the three combinations, respectively (Table 6).

**Table 6:** Synergy testing results for three combinations (Monolaurin plus ampicillin, Monolaurin plus amoxicillin, and Monolaurin plus piperacillin) against MRSA isolates

Combination		Effects			
		**Synergism**		In-difference N (%*)	Antagonistic N (%*)
		Synergistic N (%*)	Partial synergy N (%*)		
Monolaurin (250µg/ml)	Ampicillin	91(88.4%)	9(8.7%)	3(2.9%)	-
	Amoxicillin	91(88.4%)	9(8.7%)	3(2.9%)	-
	Piperacillin	74(71.8%)	26(25.3%)	3(2.9%)	-
Monolaurin (500µg/ml)	Ampicillin	8(7.8%)	83(80.6%)	12(11.6%)	-
	Amoxicillin	8(7.8%)	83(80.6%)	12(11.6%)	-
	Piperacillin	8(7.8%)	83(80.6%)	12(11.6%)	-

%* was correlated to total no. of MRSA isolates (*n* = 103)

For MSSA isolates, the combination of 250 µg/ml monolaurin with ampicillin,and amoxicillin exhibited synergism in 83.3%and partial synergy in 16.7% while, synergism in 83.3% and additive in 16.7% for monolaurin with piperacillin, respectively. Using 500 µg/ml monolaurin with ampicillin, amoxicillin and piperacillin showed partial synergy 83.3% and indifference in 16.7% of the total isolates in the all combinations, respectively (Table 7).

**Table 7:** Synergy testing results for three combinations (Monolaurin plus ampicillin, Monolaurin plus amoxicillin, and Monolaurin plus piperacillin) against MSSA isolates

Combination		Effects				
		**Synergism**		**Additive**	In-difference N (%*)	Antagonisti N (%*)
		Synergistic N (%*)	Partial synergy N (%*)			
Monolaurin (250µg/ml)	Ampicillin	10(83.3%)	2(16.7%)	-	-	-
	Amoxicillin	10(83.3%)	2 (16.7%)	-	-	-
	Piperacillin	10(83.3%)	-	2(16.7%)	-	-
Monolaurin (500µg/ml)	Ampicillin	-	10(83.3%)		2(16.7%)	-
	Amoxicillin	-	10(83.3%)		2(16.7%)	-
	Piperacillin	-	10(83.3%)		2(16.7%)	-

%* was correlated to total no. of MRSA isolates (*n* = 12)

### Time killing assay

**Fig. 4:**
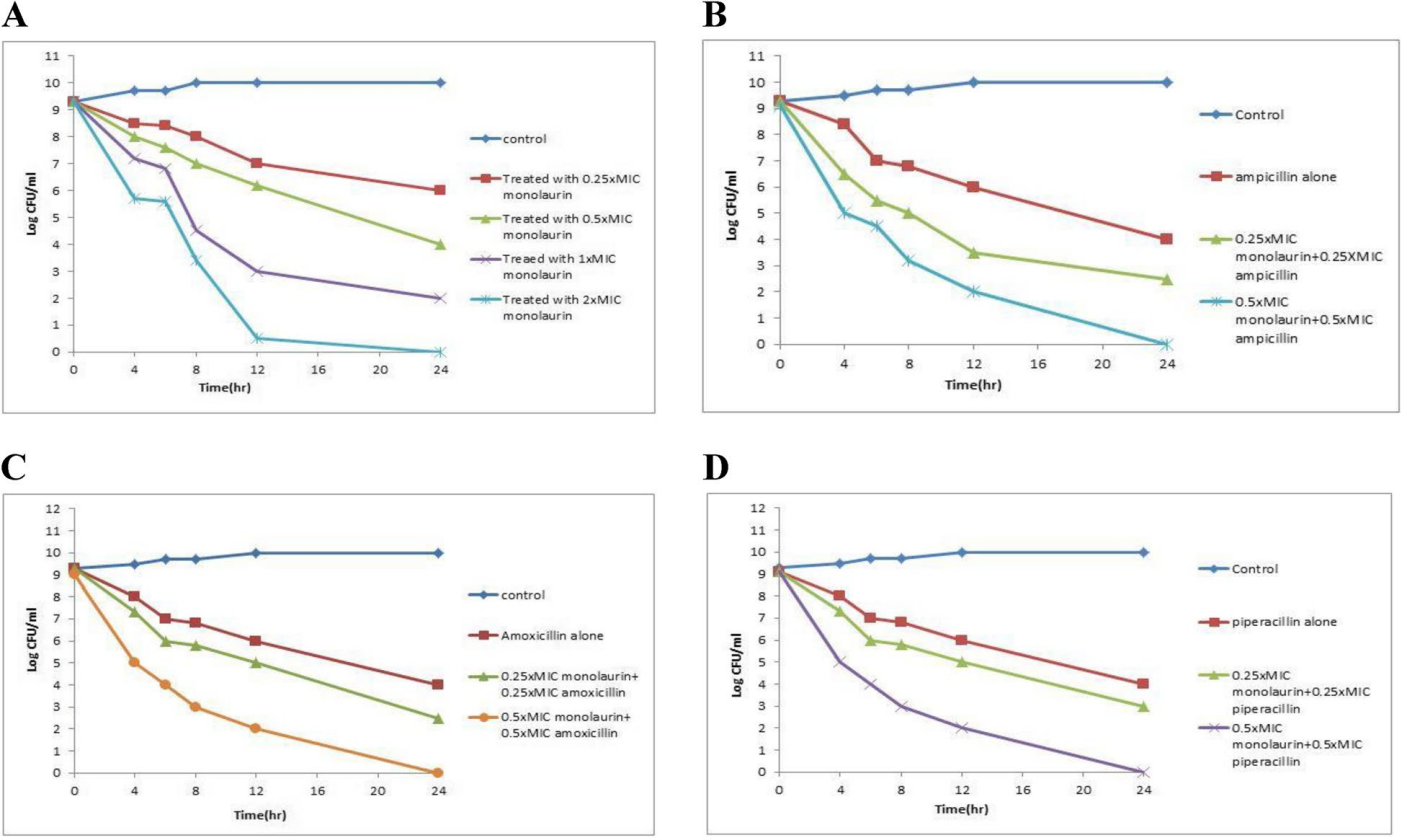
Time killing curve of S.aureus; **A** The antibacterial effect of monolaurin alone; **B** The antibacterial effect of ampicillin alone and in combination with monolaurin against S.aureus (log CFU/ml); **C** The antibacterial effects of amoxicillin alone and in combination with monolaurin against S.aureus (log CFU/ml); **D** The antibacterial effects of piperacillin alone and in combination with monolaurin against *S.aureus* (log CFU/ml) during 24 h incubation at 37 °C

To confirm the synergistic effects of monolaurin and the selected antibiotics on *S.aureus*, a time-kill assay was performed. Since most *S.aureus* isolates had MIC values of 1000 µg/mL, 10 *S.aureus* strains with that MIC were chosen, and the mean value of their results was determined. Monolaurin showed dose-dependent bactericidal activity against *S.aureus* in time-kill experiments (Fig. 4a). 10 *S.aureus* strains with that MIC were chosen, and the mean value of their results was determined. Monolaurin showed dose-dependent bactericidal activity against *S.aureus* in time-kill experiments (Fig. 4a). Bactericidal synergism was observed at 0.25 XMIC (3.3 log_10_, 4.3 log_10_ and 6.3 log_10_reduction at 8, 12 and 24 h) and 0.5XMIC (5.9 log_10_, 7.1 log_10_ and 9.1 log_10_ reduction at 8, 12 and 24 h) for monolaurin combined with ampicillin (Fig. 4b). Synergism was also observed at 0.25 × MIC (3.5 log_10_, 4.3 log_10_ and 6.8 log10 reduction at 8, 12 and 24 h, respectively) and at 0.5XMIC (6 log_10_, 7 log_10_ and 9 log_10_ reduction at 8, 12 and 24 h, respectively) for monolaurin combined with amoxicillin (Fig. 4c). The combination of monolaurin and piperacillin showed synergism at 0.25 × MIC (3.5 log10, 4.3 log_10_ and 6.3 reduction at 8, 12 and 24 h) and at 0.5 × MIC (6.1 log_10,_ 6.6 log_10_ and 9.1 log_10_ reduction at 8, 12 and 24 h) (Fig. 4d). These combinations displayed the highest antibacterial performance against *S.aureus* compared to control. In general, the results of the time-kill assay were compatible with the FIC method.

## Discussion

It is now a worldwide issue that human pathogenic microorganisms have evolved drug resistance. The spread of *S.aureus* in hospital and community settings has had a significant effect on worldwide public health [30]. Since the current medicines used to treat these resistant bacteria are no longer effective, it is vital to find new alternatives. Natural products derived from medicinal plants have shown a variety of biological activities in the biomedical field during the past few decades, including their antibacterial activity against different drug resistant microorganisms. More encouragingly, certain natural compounds may be able to make the target bacteria receptive to antibiotics once more by reversing the bacterial resistance to them [31]. This research was done to find and define the antibacterial effect as well as the possible synergistic combination between certain beta lactam antibiotics and potential antibacterial compound, monolaurin previously found in Coconut oil that was effective against *S.aureus* [16].

This study focused on the beta lactam family of antibiotics since they are still among the most frequently prescribed medication classes, but their effectiveness is constrained by the rise of bacteria with a variety of resistance mechanisms [32].

In recent years, *S.aureus* has become resistant to both new and traditional antibiotics. Thus, treatment of antibiotic resistant bacteria represents a therapeutic problem. The antibiogram of the studied *S.aureus* strains revealed that linezolid and imipenem were the most effective antibiotic against *S.aureus* (2% and 3% resistance rate) followed by vancomycin (4.35% Resistance rate) and chloramphenicol (13.9% Resistance rate). *S.aureus* showed complete resistance to ampicillin/sulbactam amoxicillin/clavulunic acid, and piperacillin/tazobactam, moderate resistance against tetracycline (57.4%), rifampicin (36.52%), ciprofloxacin (34.8%) and levofloxacin (34.8%) and gentamicin (33.9%). According to Vu et al. [33], 89% of *S.aureus* isolates were penicillin resistant, 37% were fluoroquinolone resistant, 41% were aminoglycoside resistant, and only 2% of the isolates were vancomycin resistant. These findings were in line with our findings. Similar findings were made by Ahmed et al. [34], who reported that only 3% of *S.aureus* strains were imipenem resistant, 100% were resistant to penicillin except for chloramphenicol and tetracycline, 72% of the isolates were resistant.

Our results were at conflicts with a research by Sonbol et al. [35], which revealed that ciprofloxacin had the lowest resistance rates (3.7% resistance) against the tested isolates. Additionally, substantial resistance rates to rifampin (57.4%) were found, which was higher than our findings for rifampin, respectively.

Infections due to methicillin-resistant *S.aureus* (MRSA) are globally getting worth inside and outside of hospitals. Cefoxitin becomes more recommended for detection of methicillin resistance in MRSA when using disk diffusion testing [36]. Out of 115 *S.aureus* samples used in this investigation, 103 (89.6%) were MRSA and 12 (10.4%) were MSSA. Our results were consistent with a study by Garoy et al. [37] whom found that 15 (19.5%) of the 82 *S.aureus* isolates were methicillin-sensitive *S.aureus* (MSSA), with 59 (72% of them) being MRSA. Also, high prevalence of MRSA isolates 81.2% was identified [38]. However, Chukwueze et al. [39] revealed that 102 of the 188 *S.aureus* isolates were methicillin-susceptible S.aureus and 86 were methicillin-resistant *S.aureus* (MSSA) respectively.

Based on information from other researchers and our own*, blaZ* gene identification by conventional PCR was considered as the gold standard for determining the presence of penicillinase in the tested *Staphylococci* isolates. Clinical and Laboratory Confirmation was another element in this choice. Standards Institute (CLSI), who claims that severe infections with *S.aureus* etiology requiring penicillin therapy should take the identification of this gene into consideration [40]. Detection of *bla*Z gene was found in 73.9% of *S.aureus* isolates. In Chicago, Similar results obtained by Wang et al. [41] A total of 196 isolates (73%) were *blaZ* positive. Also, our results were in accordance with the recent literature, with values of 87% and 92% [42, 43]. In Bulgaria, all tested *S.aureus* were harboured *blaZ* gene (100%).This result seems higher than our results [44].

Monolaurin's MIC for *S.aureus* was ranged from 250 to 2000 µg/ml. Similar studies reported that 1-monolaurin can prevent the growth of *S.aureus* at different concentrations, even at the lowest concentration of 100 µg/ml [45] and 500 µg/ml [46]. Monolaurin had MICs of 100 and 250 µg/ml against *S.aureus* ATCC 25923 and ATCC 1885, respectively [47, 48]. Furthermore, a comparable study on the antibacterial activity of monolaurin and lauric acid was reported by Batovska et al. [49] who demonstrated that monolaurin had relatively greater inhibitory capabilities than lauric acid against *Staphylococcus.epidermidis, Streptococcus.pyogenes, Listeria.monocytogenes, Corynebacterium.diphtheria and Bacillus.cereus* with the MIC values of 31.25, 31.25, 62.5, 62.5, and 125 µg/ml, respectively.

There have also been several reports of monolaurin's inhibitory mechanism against Gram-positive bacteria. The typical antibacterial target locations have been extensively investigated. Gram-positive bacteria's cell wall is their outermost layer. It is a crucial organelle that helps to keep the cell's structure intact and hinders the entry of external substances. Damage to the cell wall might potentially result in decreased cellular activity and metabolic disturbance brought on by invading foreign substances, which would result in cell death [50]. This was confirmed through scanning electron microscopy. SEM analysis revealed that the cells treated with monolaurin showed a morphological alteration in the form of cell elongation and swelling when compared to the control. Similar study demonstrated changes in cell activity and morphology of *S.aureus* upon using monolaurin [51].

Upon studying gene expression using real-time polymerase chain reaction (PCR), we can often investigate changes (increases or decreases) in the expression of a particular gene via measuring the amount of the gene-specific transcript. We performed gene analysis to confirm how monolaurin can affects the β-lactam resistance gene (*blaZ*). The expression of *blaZ* was significantly inhibited in tested isolates in a dose-dependent manner when they were treated with sub-MIC 250 and 500 µg/ml of monolaurin. Our results were convenient with Brown-Skrobot et al. [52] who revealed that the inhibition of beta-lactamase production can be attributed to the reduction in expression of the gene which encodes this protein (*bla*Z), i.e., the prevention of transcription of the gene through inhibition of signal transduction by glycerol monolaurate ("GML").

There were relatively few treatment choices available because of the decreased effectiveness of recently developed antibiotics and the unfavorable modifications that arise from using "old" medications. Combinatorial therapy between antibiotics and other compounds (e.g., natural product-derived) is suggested as an effective approach to help in resolving the issue of antibiotic resistance, cellular toxicity and the need for long-term therapies with the current antibiotics [53, 54]. In this present study, the combination of antibiotics with monolaurin was undertaken with the objective of enhancing their antibacterial efficacy, overcoming resistance, and diminishing both the cost and duration of antimicrobial therapy. As seen in Tables 2, 3, 4 and 5, there was a considerable reduction in the previous MICs when comparing the MIC values of antibiotic monotherapy and combination antibiotics with monolaurin.

The combinations were also investigated to assess their synergistic, indifferent, additive, or antagonistic effects through FICI determination. Employing 250 and 500 µg/ml of monolaurin in various combinations with antibiotics (ampicillin, amoxicillin, and piperacillin) against MRSA isolates demonstrated synergism rates of 97.1%, 97.1% and 88.4%, and in difference rates of 2.9% as well as 11.6%, respectively. For MSSA, combinations of 250 µg/ml monolaurin with antibiotics (ampicillin, amoxicillin, and piperacillin) demonstrated synergism rates of 100%, 100%, and 83.3%, respectively. Furthermore, combinations of 500 µg/ml monolaurin with antibiotics (ampicillin, amoxicillin, and piperacillin) exhibited synergism rates of 83.3% and in-differences of 16.7%, respectively. The time killing assay for monolaurin's antibacterial activity alone and in combination with antibiotics against *S.aureus* was illustrated in Fig. 5. The results showed that monolaurin had synergistic activity and significantly reduced the bacterial count when compared with control. Our results were agreed with Preuss et al. [55] who stated that monolaurin, alone or combined with antibiotics, might be useful in the prevention and treatment of severe bacterial infections, especially those that are antibiotic resistant. Previous reports have documented the synergistic benefits of natural products in combination with antibiotics against microbial pathogens [56–58]. Moreover, it has been demonstrated that using multiple antimicrobials together can boost their antibacterial effects while also lowering the dosages of each antimicrobial that are needed [59].

We have identified a new potential therapy against *S.aureus* consisting of a combination of clinically approved antibacterial drugs such as monolaurin and subclasses of β-lactam compounds, all targeting cell-wall synthesis: This treatment incorporates components from two different approaches: (i) combining drugs to increase antibiotic potency through synergy and (ii) use of combination to suppress resistance evolution.

## Conclusion

The goal of the current investigation was to determine if monolaurin alone or in combination with β-lactam antibiotics had any antibacterial effects on *S.aureus*. We tested the antibacterial efficacy and synergy of monolaurin in combination with β-lactam antibiotics against *S.aureus* isolates for the first time. The findings suggested that monolaurin has an efficient antibacterial action and can reduce *blaZ* expression. Consequently, the mixture of might be regarded as a unique and promising antibacterial mixture. The combination use can lessen the dosage of each antibacterial substance needed and slow the emergence of antibiotic resistance.

### Supplementary Information

**Supplementary Material 1.**

**Supplementary Material 2.**

**Supplementary Material 3.**

**Supplementary Material 4.**

**Supplementary Material  5.**

**Supplementary Material 6.**

## Data Availability

All data generated or analyzed during this study are included in this article and its Supplementary information file.

## Abbreviation

MRSA: Methicillin resistant *Staphylococcus aureus*
MSSA: Methicillin-resistant *Staphylococcus aureus*
MDR: Multi-drug resistant
FDA: Food and Drug Administration
GML: Glycerol monolaurate
TSB: Trypticase soy broth
CLSI: Clinical laboratory standard institute
β-lactam antibiotics: Beta-lactam antibiotics
MIC: Minimum inhibitory concentration
MHB: Mueller-Hinton Broth
µg/ml: Microgram per milliliter
CFU/ml: Colony forming unit per milliliter
FIC: Fractional inhibitory concentration
PCR: Polymerase chain reaction
Bp: Base pair
RT-PCR: Real time -polymerase chain reaction
CT: Cycle threshold
SEM: Scanning electron microscope
*bla*Z: Beta-lactamase Z gene
ST: *Staphylococcus aureus*
