# Supplementary material for: Novel synergistic interactions between monolaurin, a mono-acyl glycerol and β lactam antibiotics against Staphylococcus aureus: an in vitro study
Source: BMC Infect Dis. 2024 Apr 8;24:379. doi: 10.1186/s12879-024-09261-9 (PMC11000382; doi:10.1186/s12879-024-09261-9)
Supplement: Supplementary file 1 — Supplementary Material 1. [file 12879_2024_9261_MOESM1_ESM.pdf]

Additional file 1

Table S1: Distribution of MRSA and MSSA in different clinical Specimens

**Type of infection**		***S. aureus* isolates**			
		**MRSA**		**MSSA**	
		**No**	**%***	**No**	**%***
**Wound infections**	Surgical wound	42	**36.52**	4	**3.48**
	Accidental wound infection	21	**18.26**	2	**1.74**
	Ulcers& Abscess	11	**9.56**	2	**1.74**
**Burns**		21	**18.26**	3	**2.61**
**Blood**		2	**1.74**	1	**0.87**
**Urinary tract infections**		3	**2.61**	0	**0**
**Chest infections**		3	**2.61**	0	**0**
**Total**		103	**89.6**	12	**10.4**

%* correlate to total no. of *S.aureus* isolates (n=115)
