# Supplementary material for: Novel synergistic interactions between monolaurin, a mono-acyl glycerol and β lactam antibiotics against Staphylococcus aureus: an in vitro study
Source: BMC Infect Dis. 2024 Apr 8;24:379. doi: 10.1186/s12879-024-09261-9 (PMC11000382; doi:10.1186/s12879-024-09261-9)
Supplement: Supplementary file 2 — Supplementary Material 2. [file 12879_2024_9261_MOESM2_ESM.pdf]

Additional file 2

Table S2: Oligonucleotide primers and probes used in SYBR Green real time PCR:

Gene	Primer sequence (5'-3')	Reference
blaZ	**F** TACAACTGTAATATCGGAGGG	**[14]**
	**R** CATTACACTCTTGGCGGTTTC	
16S rRNA	**F** CCTATAAGACTGGGATAACTTCGGG	**[19]**
	**R** CTTTGAGTTTCAACCTTGCGGTCG	
