# Supplementary figures and images for: Novel synergistic interactions between monolaurin, a mono-acyl glycerol and β lactam antibiotics against Staphylococcus aureus: an in vitro study
Source: BMC Infect Dis. 2024 Apr 8;24:379. doi: 10.1186/s12879-024-09261-9 (PMC11000382; doi:10.1186/s12879-024-09261-9)

**Additional file 3**

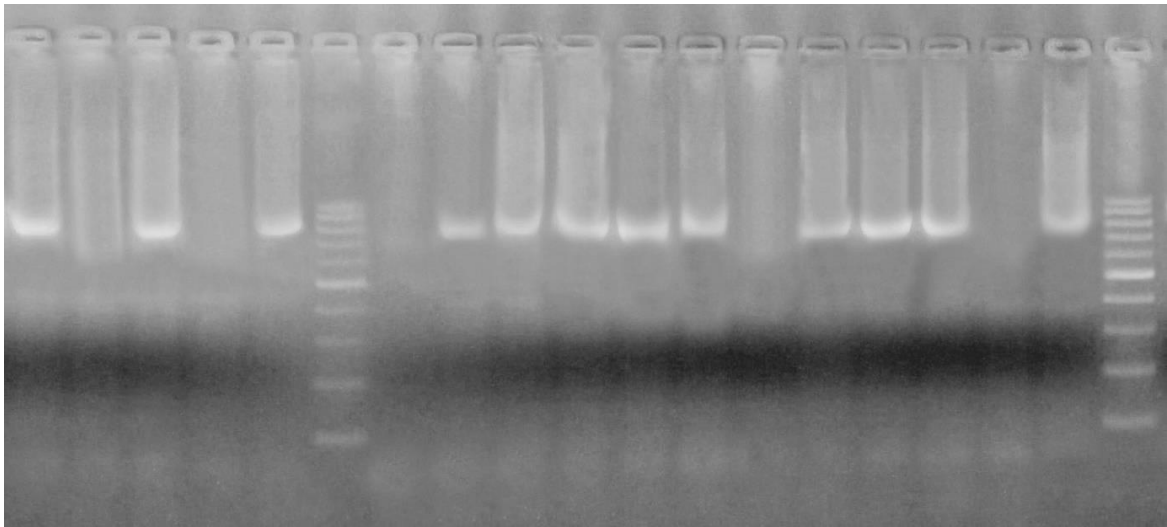

**Fig. S1 PCR detection of *blaZ* gene**

Supplement: Supplementary file 3 — Supplementary Material 3. [file 12879_2024_9261_MOESM3_ESM.pdf]

## Additional file 4

**Fig. S2**

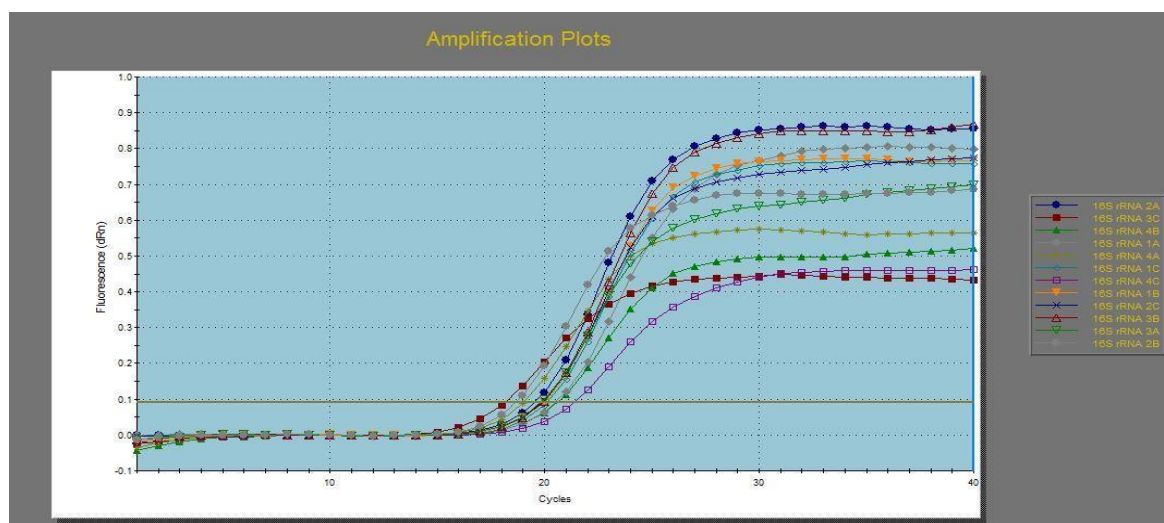

**Amplification curve of 16s rRNA**

Supplement: Supplementary file 4 — Supplementary Material 4. [file 12879_2024_9261_MOESM4_ESM.pdf]

## Additional file 5

**Fig.S3**

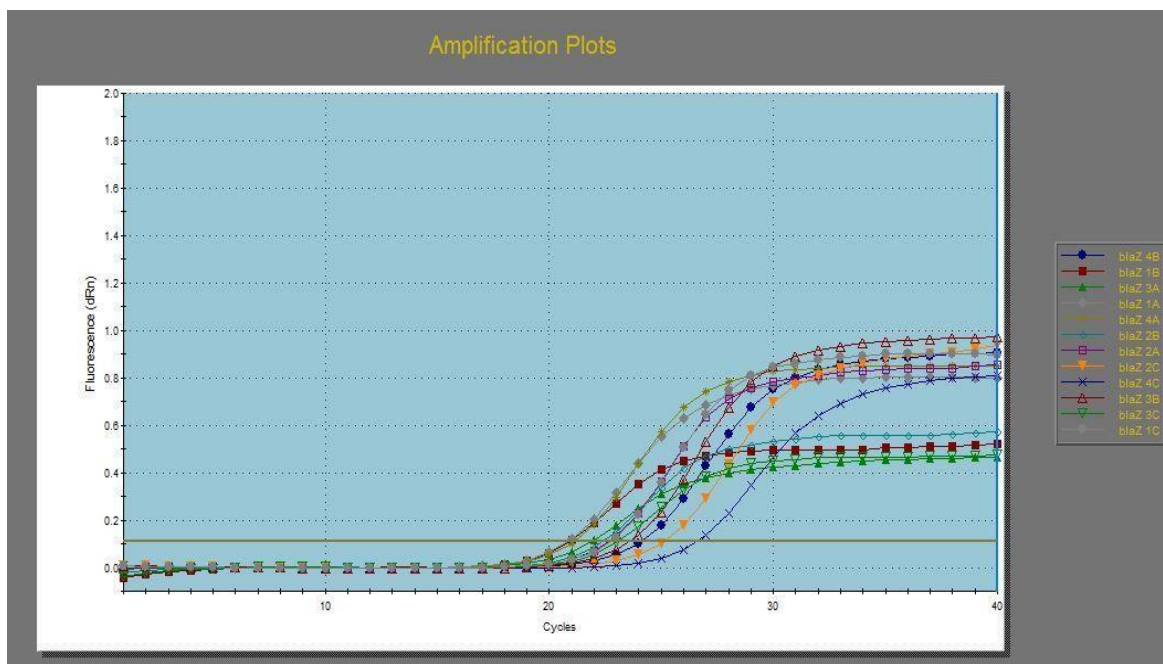

**Amplification curve of *blaZ* gene**

Supplement: Supplementary file 5 — Supplementary Material 5. [file 12879_2024_9261_MOESM5_ESM.pdf]
