# Supplementary material for: Novel synergistic interactions between monolaurin, a mono-acyl glycerol and β lactam antibiotics against Staphylococcus aureus: an in vitro study
Source: BMC Infect Dis. 2024 Apr 8;24:379. doi: 10.1186/s12879-024-09261-9 (PMC11000382; doi:10.1186/s12879-024-09261-9)

Additional file 6

Table S3: Gene expression of S.aureus upon using monolaurin at sub-MICs concentrations:

**Sample ID.**	**Sample No.**	***blaZ***	***16S rRNA***	**Fold change**	**Fold change reduction**	**%***
ST1(control)	1A	20.15	21.08	-	-	-
ST1(Treated with 250µg/ml monolaurin)	1B	19.72	21.32	0.6285	0.3715	**37.15**
ST1 (Treated with 500µg/ml monolaurin)	1C	20.11	22.85	0.2852	0.7148	**71.48**
ST2 (control)	2A	19.47	22.19	-	-	**-**
ST2(Treated with 250µg/ml monolaurin)	2B	18.54	22.09	0.5625	0.4375	**43.75**
ST2(Treated with 500µg/ml monolaurin)	2C	19.62	25.10	0.1476	0.8524	**85.24**
ST3(control)	3A	19.51	22.03	-	-	**-**
ST3(Treated with 250µg/ml monolaurin)	3B	19.70	23.17	0.5176	0.4824	**48.24**
ST3(Treated with 500µg/ml monolaurin)	3C	18.33	23.12	0.2073	0.7927	**79.27**
ST4(control)	4A	19.30	21.74	-	-	**-**
ST4(Treated with 250µg/ml monolaurin)	4B	20.19	23.55	0.5285	0.4715	**47.15**

ST4(Treated with 500µg/ml monolaurin)	4C	21.41	26.92	0.1191	0.8809	**88.09**

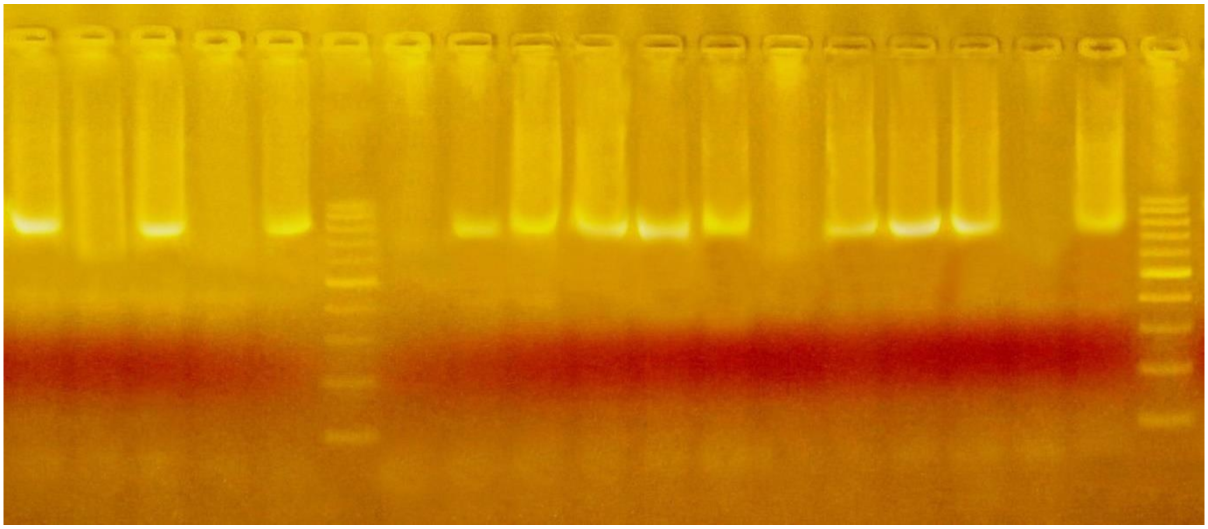

Supplement: Supplementary file 6 — Supplementary Material 6. [file 12879_2024_9261_MOESM6_ESM.pdf]
